# GRK2 regulates GLP-1R-mediated early phase insulin secretion in vivo

**DOI:** 10.1186/s12915-021-00966-w

**Published:** 2021-03-03

**Authors:** Alba C. Arcones, Rocío Vila-Bedmar, Mercedes Mirasierra, Marta Cruces-Sande, Mario Vallejo, Ben Jones, Alejandra Tomas, Federico Mayor, Cristina Murga

**Affiliations:** 1 Departamento de Biología Molecular and Centro de Biología Molecular Severo Ochoa (CBMSO) UAM-CSIC; Instituto de Investigación Sanitaria Hospital Universitario La Princesa; CIBER de Enfermedades Cardiovasculares (CIBERCV), UNIVERSIDAD AUTONOMA DE MADRID and Instituto de Salud Carlos III, Madrid, Spain; 2 Departamento de Ciencias Básicas de la Salud, Facultad de Ciencias de la Salud, Universidad Rey Juan Carlos (URJC), Madrid, Spain; 3 Instituto de Investigaciones Biomédicas Alberto Sols (CSIC-UAM); Centro de Investigación Biomédica en Red de Diabetes y Enfermedades Metabólicas Asociadas (Ciberdem), Madrid, Spain; 4 Section of Investigative Medicine, Imperial College London, London, W12 0NN UK; 5 Section of Cell Biology and Functional Genomics, Imperial College London, London, W12 0NN UK

**Keywords:** Insulin signaling, Incretin, G protein-coupled receptor kinase 2 (GRK2), Glucagon-like peptide 1 (GLP-1), β-arrestin, Granule dynamics

## Abstract

**Background:**

Insulin secretion from the pancreatic β-cell is finely modulated by different signals to allow an adequate control of glucose homeostasis. Incretin hormones such as glucagon-like peptide-1 (GLP-1) act as key physiological potentiators of insulin release through binding to the G protein-coupled receptor GLP-1R. Another key regulator of insulin signaling is the Ser/Thr kinase G protein-coupled receptor kinase 2 (GRK2). However, whether GRK2 affects insulin secretion or if GRK2 can control incretin actions in vivo remains to be analyzed.

**Results:**

Using GRK2 hemizygous mice, isolated pancreatic islets, and model β-cell lines, we have uncovered a relevant physiological role for GRK2 as a regulator of incretin-mediated insulin secretion in vivo. Feeding, oral glucose gavage, or administration of GLP-1R agonists in animals with reduced GRK2 levels (GRK2+/− mice) resulted in enhanced early phase insulin release without affecting late phase secretion. In contrast, intraperitoneal glucose-induced insulin release was not affected. This effect was recapitulated in isolated islets and correlated with the increased size or priming efficacy of the readily releasable pool (RRP) of insulin granules that was observed in GRK2+/− mice. Using nanoBRET in β-cell lines, we found that stimulation of GLP-1R promoted GRK2 association to this receptor and that GRK2 protein and kinase activity were required for subsequent β-arrestin recruitment.

**Conclusions:**

Overall, our data suggest that GRK2 is an important negative modulator of GLP-1R-mediated insulin secretion and that GRK2-interfering strategies may favor β-cell insulin secretion specifically during the early phase, an effect that may carry interesting therapeutic applications.

**Supplementary Information:**

The online version contains supplementary material available at 10.1186/s12915-021-00966-w.

## Background

Insulin is the major anabolic hormone controlling metabolic homeostasis. Consequently, the pancreatic β-cell is poised to rapidly adapt the rate of insulin secretion to fluctuations in blood glucose concentration by a complex array of regulatory mechanisms [1]. Upon increased blood glucose levels, the canonical pathway of insulin secretion is activated following glucose entry in the β-cell through the GLUT2 transporter. Intracellular glucose undergoes glycolysis, increasing the ATP/ADP ratio and leading to closure of K^+^/ATP-dependent channels. This results in membrane depolarization and activation of voltage-dependent Ca^2+^ channels, increasing intracellular calcium concentration and triggering pulsatile insulin secretion [1]. Besides, different extracellular mediators acting via G protein-coupled receptors (GPCRs) such as incretins, adrenergic, and muscarinic agonists, as well as signals from nutrient receptors, converge on the β-cell to finely modulate insulin release [2–5].

Insulin secretion occurs in a biphasic manner. The so-called early phase takes place during the first 10–15 min after feeding and represents the release of insulin already stored in granules. The more sustained late phase requires new synthesis and processing of insulin and can be modulated by the decrease in systemic glucose caused by the effects of insulin in tissues such as the muscle and the liver [6, 7]. After insulin synthesis and granule maturation, most insulin granules (75–95%) are stored within the β-cell cytoplasm, constituting the releasable pool (RP). A cortical actin network acts as a physical barrier between the RP and the readily releasable pool (RRP) of granules, which are primed at the cell membrane. This allows for rapid calcium-dependent fusion and insulin release from the RRP, which is especially relevant during the early phase [8–11].

The enteroendocrine system is an important modulator of early and late phase insulin secretion in the post-prandial state. Specialized nutrient-sensing cells respond to food intake by releasing peptide hormones into the circulation, which act either directly on the β-cell, or indirectly via neural relays, and may account for 60–70% of total insulin release in healthy human subjects [7, 12]. Of special relevance is the glucagon-like peptide 1 (GLP-1), secreted by the L-cells of the small and large intestine following post-translational processing of the proglucagon gene. In β-cells, GLP-1 acts on the GLP-1 receptor (GLP-1R), a Gαs-coupled class B GPCR that triggers a transient increase in cAMP, thus activating protein kinase A (PKA) and/or exchange protein directly activated by cAMP 2 (EPAC2) effectors. These signaling pathways potentiate glucose-induced closure of the K^+^/ATP channels and promote further β-cell depolarization and Ca^2+^ influx, ultimately leading to augmented insulin secretion [13–16]. The GLP-1R-EPAC2 pathway is also implicated in the potentiation of the early phase of insulin release by regulating insulin granule maturation, trafficking, and exocytosis [7, 17–19]. These processes are also modulated by GLP-1 stimulation of PKA, although its contribution to granule dynamics is less well established [20, 21]. The induction of insulin release only in the presence of high glucose (thus avoiding the risk of hypoglycaemia) and modulation of the early phase of insulin secretion account for the increasing use of GLP-1 mimetics in the treatment of type 2 diabetes (T2D) [16, 22–25].

G protein-coupled receptor kinase 2 (GRK2), a product from the *adrbk1* gene, is a Ser/Thr kinase classically known for its role in the regulation of GPCRs. GRK2 phosphorylates the active form of GPCRs, thus prompting the recruitment of β-arrestins and the uncoupling of G proteins. This leads to internalization of GPCRs and also triggers β-arrestin-dependent signals [26, 27]. Besides this canonical role, GRK2 is able to interact with other non-GPCR partners in a kinase-dependent or independent manner [28–31]. In particular, GRK2 acts as a negative regulator of insulin signaling by different mechanisms taking place downstream or at the level of the insulin receptor [26, 27, 32]. We have previously reported that GRK2 levels increase in several key metabolic tissues in experimental conditions promoting insulin resistance (IR) and that GRK2+/− mice (the use of GRK2−/− animals is not experimentally possible since they die during embryogenesis [33]) are protected against high-fat diet (HFD)-induced obesity and IR. Also, reduction of GRK2 levels in mice with an already established obese phenotype is able to revert the obese and IR state [27, 34]. These data point to the potential for beneficial effects of reducing GRK2 in key metabolic tissues such as the liver, muscle, and white adipose tissue. Interestingly, in rodent models and also in humans, the development of IR in different tissues promotes a compensatory effect in the pancreas that initially leads to hyperinsulinemia and ultimately triggers β-cell failure [35, 36].

In this report, we have addressed the potential role of GRK2 levels in pancreatic functionality in non-IR conditions using GRK2 hemizygous mice and β-cell models. Our results reveal that low levels of GRK2 enhance GLP-1R-mediated insulin secretion in vivo and in isolated islets. We also show that GLP-1R activation promotes GRK2 association and β-arrestin recruitment in a GRK2-dependent manner. In vivo, reduced GRK2 levels potentiate insulin release, particularly from the RRP of insulin granules, in response to the GLP-1R agonist Exendin-4. We also find that GRK2 modulates insulin secretion from the β-cell specifically during the early phase, which may have potential therapeutic implications.

## Results

### GRK2 protein expression in the pancreas is restricted to the pancreatic islets

**Fig. 1:**
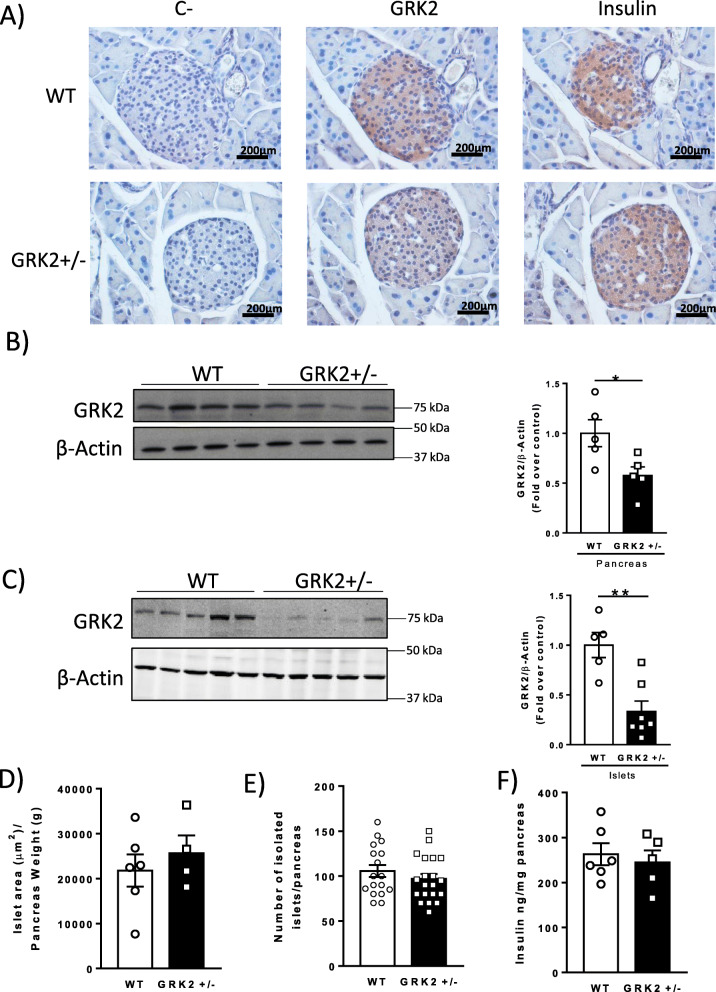
Specific localization of GRK2 in the pancreatic islet and expression levels of GRK2 protein in the pancreas of WT and GRK2+/− mice. Representative photomicrographs showing the immunohistochemical staining of serial pancreatic sections using antibodies against GRK2 or insulin as an islet marker, counterstained with hematoxylin (magnification × 40; scale bar 0.2 mm). Incubations without primary antibody are performed as a negative control (**a**). Whole pancreatic tissue lysates, WT *n* = 5, GRK2+/− *n* = 5 (**b**) or isolated islets lysates, WT *n* = 5, GRK2+/− *n* = 7 (**c**) were subjected to Western blot analysis and probed with antibodies against GRK2 and β-actin. Stereological analysis of the pancreas analyzing three pancreatic sections separated 0.4 mm (each whole section was imaged, with an average 30 islets detected per section: total 565 islets were counted in WT mice (average of 94 islets/mice in three sections) and 407 islets in GRK2+/− mice (average of 101 islets/mice in three sections), non statistically different by *t* test), islet mass was quantified measuring insulin-positive area in WT *n* = 6 and GRK2+/− *n* = 4 (**d**). Number of isolated islets per digested pancreas, WT *n* = 17, GRK2+/− *n* = 20 (**e**). Total pancreatic insulin content measured in acidic-extracts of pancreata by ELISA, WT *n* = 6 GRK2+/− *n* = 5 (**f**). Means ± SEM data are represented, statistical analysis was performed using unpaired *t* test. *p < 0.05, **p<0.01

The pancreas is a heterogeneous organ constituted by the exocrine pancreas (acinar and duct cells) and the endocrine pancreas (the islets) [37, 38]. In order to characterize the distribution of GRK2 protein expression in the pancreas, we performed immunohistochemical (IHC) detection in pancreatic sections of WT and GRK2 hemizygous (GRK2+/−) mice. GRK2 protein was restricted to the pancreatic islets, and no GRK2 staining was found in exocrine pancreatic tissue (Fig. 1a and Additional File 1: Supplementary Figure 1). GRK2+/− mice display circa 50% of GRK2 protein levels compared with their WT littermates as quantified by Western blot both in lysates from whole pancreata (Fig. 1b) and from isolated islets (Fig. 1c). No apparent differences were revealed in islet mass by histological quantification of serial sections of the pancreas from WT and GRK2+/− mice (Fig. 1d), in islet numbers obtained from these animals (Fig. 1e) or in total insulin levels as confirmed by acidic extraction from whole pancreata (Fig. 1f). These data reveal that GRK2 levels do not influence islet mass or pancreatic insulin content.

### GRK2+/− mice display increased insulin release during the early but not late phase of insulin secretion

**Fig. 2:**
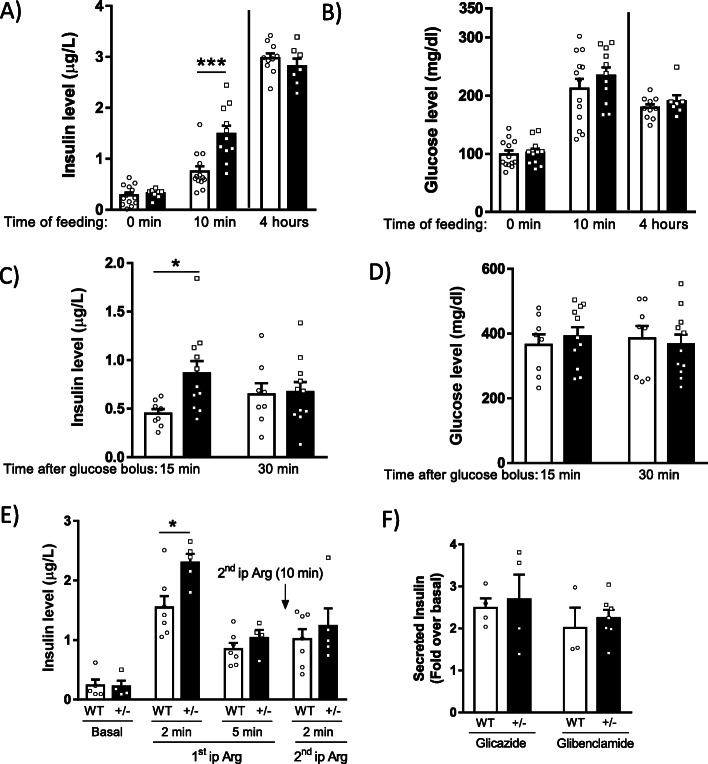
Increased early phase insulin secretion and RRP size in GRK2+/− mice. Insulin (**a**) and glucose (**b**) were measured after feeding animals for 10 min (early phase insulin secretion) or 4 h (late phase) (same animals were used to assess insulin and glucose levels, WT *n* = 13, 13, 10 and GRK2+/− *n* = 11, 11, 7; for 0, 10 min, and 4 h, respectively). oGTT (2 g/kg) was performed in WT and GRK2+/− mice, and insulin (**c**) and glucose levels (**d**) were assessed in serum samples 15 min (early phase) and 30 min (late phase) after a glucose gavage (same animals were used to assess insulin and glucose levels, WT *n* = 7, GRK2+/− *n* = 10). To explore the status of different pools of insulin granules, mice were injected ip with 1 g/kg arginine (1st ipArg; measured at 2 and 5 min) to elicit insulin secretion from the RRP. A second arginine injection 10 min later (2nd ipArg; measured 2 min later) reveals effects in replenishing the RRP from the RP. In both cases, insulin concentrations in serum are shown in the graph (WT *n* = 5, 7, 7, 7; GRK2 +/− *n* = 4, 5, 4, 5 for 0, 2, 5 min and 2 min after 2nd ip Arg, respectively) (**e**). Mice were injected ip with the sulfonylureas glicazide (10 mg/kg) or glibenclamide (5 mg/kg). Insulin levels were measured at 0 and 15 min and fold increase in serum insulin levels is shown (glicazide: WT *n* = 4, GRK2+/− *n* = 4; glibenclamide: WT *n* = 3, GRK2+/− *n* = 7) (**f**). Means ± SEM data are represented, WT: White bars, GRK2+/−: Black bars, statistical analysis was performed by 1-way ANOVA followed by Bonferroni’s post hoc test *p < 0.05; ***p < 0.01

Given the specific localization of pancreatic GRK2 inside the islets, we set out to determine whether GRK2 expression could have an impact on islet function, first by investigating early and late phase insulin secretion in response to feeding in WT and GRK2+/− animals. GRK2+/− mice showed higher plasma insulin concentrations than WT littermates 10 min (early phase) but not 4 h (late phase) after ad libitum feeding (Fig. 2a). There were no differences in blood glucose levels between genotypes (Fig. 2b), making glucose availability an unlikely explanation for the observed disparity in insulin release.

The aforementioned data suggested that lower levels of GRK2 in the pancreas favor secretion of insulin after feeding. Apart from the insulinogenic effect caused by an increase in blood glucose, feeding also activates different signaling networks in the central nervous system, as well as incretin production by the gut and signaling by nutrient receptors [1]. Thus, we performed oral glucose tolerance tests (oGTTs) to promote both incretin secretion and increase blood glucose levels to synergistically trigger insulin secretion by the β-cell [39]. GRK2+/− mice displayed a markedly increased insulin secretion in oGTTs compared to WT littermates (Fig. 2c). This occurred only during the early (15 min) but not in the late (30 min) phase, even when the same concentration of gavage-elicited plasma glucose was observed (Fig. 2d), suggesting that the increased insulin secretion observed in GRK2+/− mice might be ascribed to incretin-mediated effects.

Biphasic insulin secretion from pancreatic β-cells is explained by the existence of two different pools of insulin secretory granules, the readily releasable pool (RRP) that is ready to be secreted during the early phase, and the releasable pool (RP), which needs to be first recruited to the plasma membrane in order to be released during the late phase [7]. To study in vivo granule distribution and its impact on insulin secretion dynamics, mice were subjected to arginine tolerance tests (ArgTTs). These tests serve to analyze the capacity of β-cells to release insulin from either the RRP or the RP pools after membrane depolarization [40, 41]. In particular, the first intraperitoneal arginine injection causes an initial depolarization wave that promotes insulin release from membrane-proximal RRP granules. A subsequent second injection some minutes later serves to analyze insulin secretion from the RP pool since it promotes mobilization of RP granules to replenish the RRP. Interestingly, GRK2+/− mice displayed increased insulin release only during the early RRP-secreting phase (Fig. 2e) but no differences were found in insulin released from RP-mobilized particles. Altogether, these data suggest that a differential distribution of stored insulin exists in GRK2+/− mice, with an increased size or priming/exocytotic efficiency of the RRP compared to WT animals, in the absence of significant differences in granule recruitment from the RP.

Overall, the increased early phase insulin secretion after ad libitum feeding and oGTT in GRK2+/− mice raises the possibility that β-cell responses to incretin hormones are modulated by GRK2 levels. Incretins are known to be implicated both in enhancing insulin secretion after an oral glucose bolus and in regulating the subcellular distribution of insulin granules mainly via EPAC2-dependent mechanisms [7, 18, 19].

Since GRK2 has been shown, in other cell types, to directly interact and inhibit the related EPAC1 isoform through phosphorylation [42], we set out to explore whether a decreased GRK2 dosage could directly influence EPAC2-dependent insulin secretion from pancreatic β-cells. We thus compared the effect of K^+^/ATP channel closing sulfonylureas (glicazide) and channel closing plus EPAC2-activating sulfonylureas (glibenclamide) in WT and GRK2+/− mice [43]. The results shown in Fig. 2f indicate, in a limited number of animals, that insulin release is potentiated to the same extent in both genotypes by sulfonylureas regardless of whether they are also directly activating EPAC2 or not. Altogether, these data argue in favor of the possibility that GRK2 does not modulate EPAC2 directly in pancreatic β-cells although additional research would be required to address this point.

### GRK2 is recruited to the activated GLP-1R in pancreatic β-cell lines and is required for β-arrestin 2 association

**Fig. 3:**
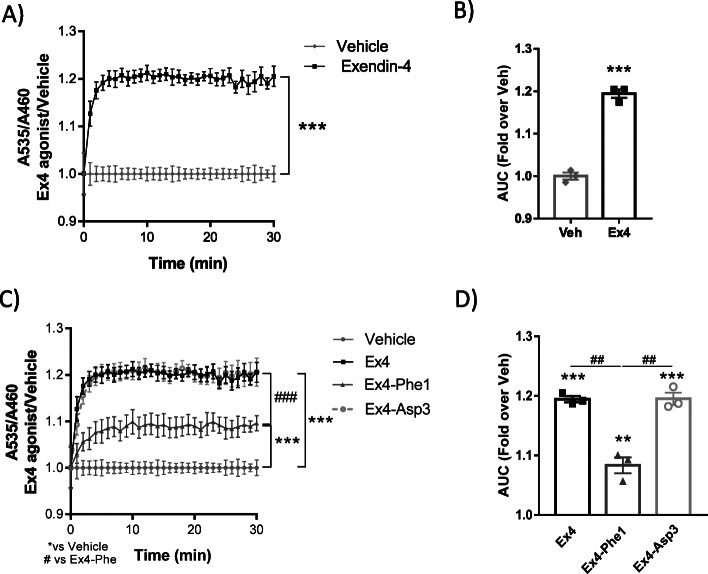
GRK2 is recruited to the activated GLP-1R and this occurs to a different extent by biased agonists. GRK2 recruitment to GLP-1R was measured by nanoBRET in INS1 832/3 GLP-1R KO cells using 100 nM of Exendin-4 (**a**). AUC of GRK2 recruitment assay by nanoBRET (**b**), *n* = 3 independent experiments, each point is an average from 3 to 4 technical replicas (**a**, **b**). The same experiment was performed in the presence of 100 nM of different Exendin-4 biased agonists: Ex4-Phe1 (Gαs-biased) and Ex4-Asp3 (β-arrestin biased) (**c**). AUC of GRK2 recruitment assay by nanoBRET (**d**). *N* = 3 independent experiments, each point is an average from 3 to 4 technical replicas. Means ± SEM data are represented, statistical analysis was performed using repeated measures two-way ANOVA (**a**, **c**) or paired one-way ANOVA (**d**) followed by Bonferroni’s post hoc test and paired *t* test (**b**), **/^##^*p* < 0.01, ***/^###^*p* < 0.001

GRK2 may be influencing incretin-driven insulin release by a mechanism that lays upstream of EPAC2, possibly at the level of the incretin receptor. We thus assessed GRK2 recruitment to activated GLP-1R and its functional effects on β-arrestin and G protein coupling using a nanoBRET-based assay in the rat pancreatic β-cell line INS-1 832/3. We observed a rapid and sustained translocation of GRK2 to the vicinity of GLP-1R after addition of Exendin-4, a well-established pharmacological agonist of this receptor [44] (Fig. 3a, b). Moreover, the recruitment of GRK2 to GLP-1R appears to be modulated by the biased signaling properties of GLP-1R ligands, as demonstrated by using previously-described Exendin-4-based biased agonists [45]. As shown in Fig. 3c, d, the Gαs-biased agonist Ex4-Phe1, known to display very reduced β-arrestin recruitment, promotes a lower association of GRK2 to GLP-1R. Conversely, the β-arrestin-biased peptide Ex4-Asp3 showed a similar recruitment of GRK2 as Exendin-4 itself. These results indicate that GRK2 is recruited to GLP-1R by Exendin-4 in β-cells and that agonists biased towards β-arrestin recruit GRK2 more efficiently than Gαs-biased ones.

**Fig. 4:**
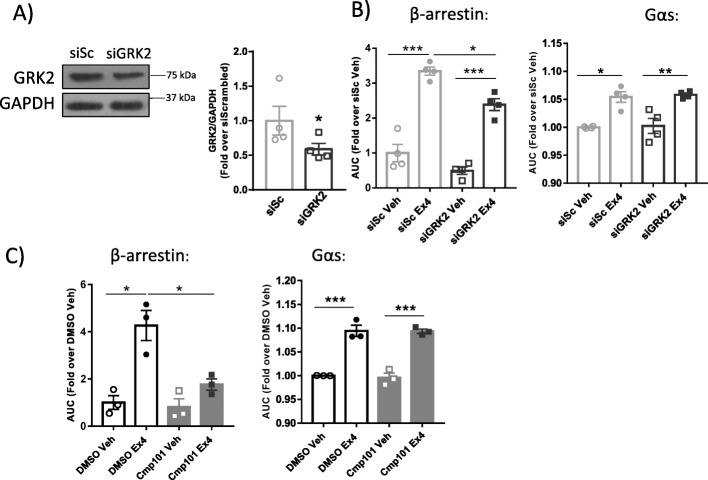
GRK2 levels and activity can modulate β-arrestin 2 association. Western blot analysis of the level of GRK2 silencing in Min6B1 cells (siSc: siRNA Scrambled; siGRK2: siRNA αAdrbk1), *n* = 4 independent experiments (**a**). AUC of β-arrestin 2 or Gαs recruitment quantified by NanoBIT and nanoBRET, respectively, for 30 min after GLP-1R activation in Min6B1 cells silenced (**b**) or inhibited (**c**) for GRK2, *n* = 4 (**b**) or 3 (**c**) independent experiments, each point is an average from 3 to 4 technical replicas. Means ± SEM data are represented. Statistical analysis was performed by paired *t* test (**a**) or paired one-way ANOVA followed by Bonferroni’s post hoc test (**b**, **c**). *p < 0.05, **p < 0.01, ***p < 0.001

To further assess this, we quantified the association of Gαs or β-arrestin 2 proteins to the activated GLP-1R using, in this latter case, the NanoBIT system (that leads to reconstitution of luciferase and light emission upon close interaction of fusion proteins) in Min6B1 β-cells with silenced or pharmacologically-inhibited GRK2. Using this system, we observed that a circa 50% reduction of GRK2 protein levels by transfecting siRNAs (Scrambled: siSc or αAdrbk1: siGRK2) (Fig. 4a) impairs β-arrestin 2 but does not change Gαs recruitment to the agonist-stimulated receptor (Fig. 4b). The same tendency was observed when GRK2 kinase activity is inhibited using the pharmacological inhibitor Compound 101 [46], which reduced the association of β-arrestin 2 but not of Gαs to the activated GLP-1R (Fig. 4c). Overall, these results put forward that GRK2 can be recruited to the activated GLP-1R in β-cell lines, where it exerts modulatory actions in β-arrestin recruitment to this receptor.

### GRK2 modulates GLP-1R-mediated insulin secretion in vivo

**Fig. 5:**
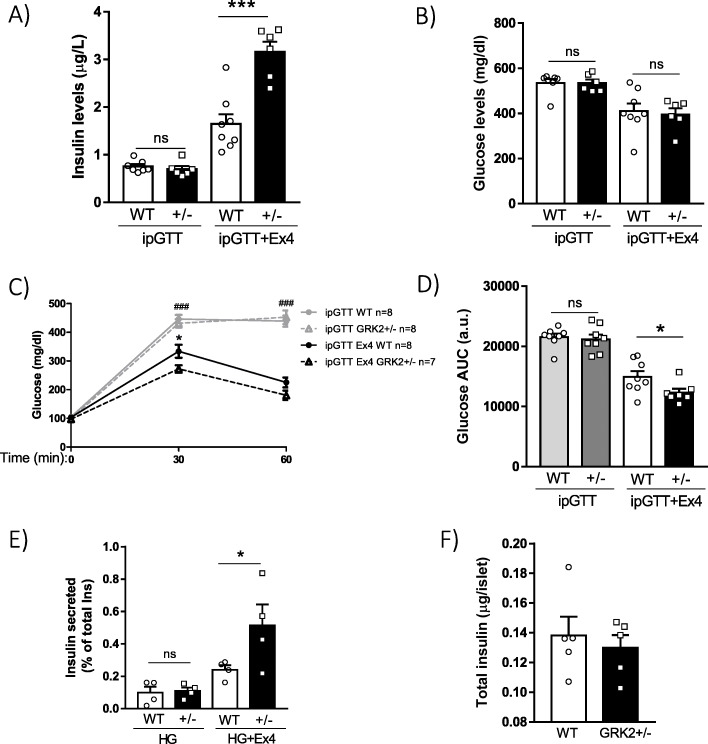
GRK2 +/− mice display increased GLP-1R-dependent insulin release in vivo and in isolated islets. Insulin (**a**) and glucose (**b**) levels were assessed in serum samples of fasted mice (basal) and 15 min after the administration of an intraperitoneal glucose bolus (ipGTT, 2 g/kg) with or without the GLP-1 analog Exendin-4 (Ex4, 5 μg/kg (for ipGTT or ipGTT+Ex4, respectively, WT *n* = 7 or 8; for GRK2+/− *n* = 6 or 6; same mice were used to assess insulin and glucose levels). Analysis of glucose levels (**c**) and bar graph representing the area under the curve (AUC) (**d**) after insulinogenic stimuli are shown; WT: *n* = 8 for ipGTT and ipGTT + Ex4 and GRK2+/− *n* = 8 for ipGTT and 7 for ipGTT + Ex4. Insulin secretion in isolated pancreatic islets stimulated with high glucose (HG, 17 mM) or high glucose with Exendin-4 (HG + Ex4 100 nM) from WT and GRK2+/− mice, expressed as % of insulin content, *n* = 4 mice (2–3 different 5-islets pool were assessed per mice as technical replicas) (**e**). Amount of total islet insulin content as obtained by acidic extraction, *n* = 5 mice (8–11 5-islets pools were assessed per mice as technical replicas) (**f**). Means ± SEM data are represented. Statistical analysis was performed using repeated measures 2-way ANOVA (**c**) or 1-way ANOVA (**a**, **b**, **d**, and **e**) followed by Bonferroni’s post hoc test and unpaired *t* test (**f**); (*) was used for comparisons between WT and GRK2+/− mice, (#) was used for comparisons between basal vs ipGTT or ipGTT vs ipGTT + Ex4 (C). *p < 0.05; *** ^###^*p* < 0.001

In order to determine whether changes in GRK2 levels could have an effect on GLP-1R-dependent enhancement of insulin secretion in vivo, we analyzed possible differences between WT and GRK2+/− mice in the potentiation of insulin release caused by Exendin-4. First, we found that the early phase insulin secretion stimulated by ip injection of glucose alone was indistinguishable in GRK2+/− animals compared to WT mice (Fig. 5a), contrary to the enhanced response observed upon feeding (Fig. 2a) or a glucose gavage (Fig. 2c), and coherent with the suggested involvement of incretins in the observed effect of GRK2 dosage.

Consistent with this notion, upon addition of Exendin-4 to the ip glucose bolus, an enhancement in insulin secretion was detected in GRK2+/− mice compared to WT (Fig. 5a). Basal and early (15 min) blood glucose levels were also similar between genotypes in the different conditions tested (Fig. 5b), but the higher insulin secretion of GRK2+/− animals in response to Exendin-4 led to a decrease in blood glucose levels that was significantly different from that of WT mice at 30 min (Fig. 5c). A similar tendency towards decreased glucose levels during the Ex4-ipGTT in these mice was suggested by the AUC analysis (Fig. 5d).

To further confirm the implication of reduced GRK2 levels particularly in the pancreas in the observed phenotype, and to avoid the possible influence of reduced GRK2 in other tissues, Exendin-4-mediated stimulation of insulin secretion was measured in pancreatic islets isolated from WT or GRK2+/− mice. We observed an increased capacity to secrete insulin upon GLP-1R activation in islets isolated from GRK2+/− animals compared to WT mice (Fig. 5e). Moreover, this enhancement in GLP-1R-dependent insulin release occurred in the absence of differences in high glucose-stimulated insulin secretion (Fig. 5e), or in total insulin (Fig. 5f) between genotypes.

In light of these results, we can conclude that GRK2 negatively regulates GLP-1R-mediated pancreatic insulin secretion and that decreasing GRK2 levels is able to boost incretin-dependent potentiation of insulin release in vivo.

## Discussion

**Fig. 6:**
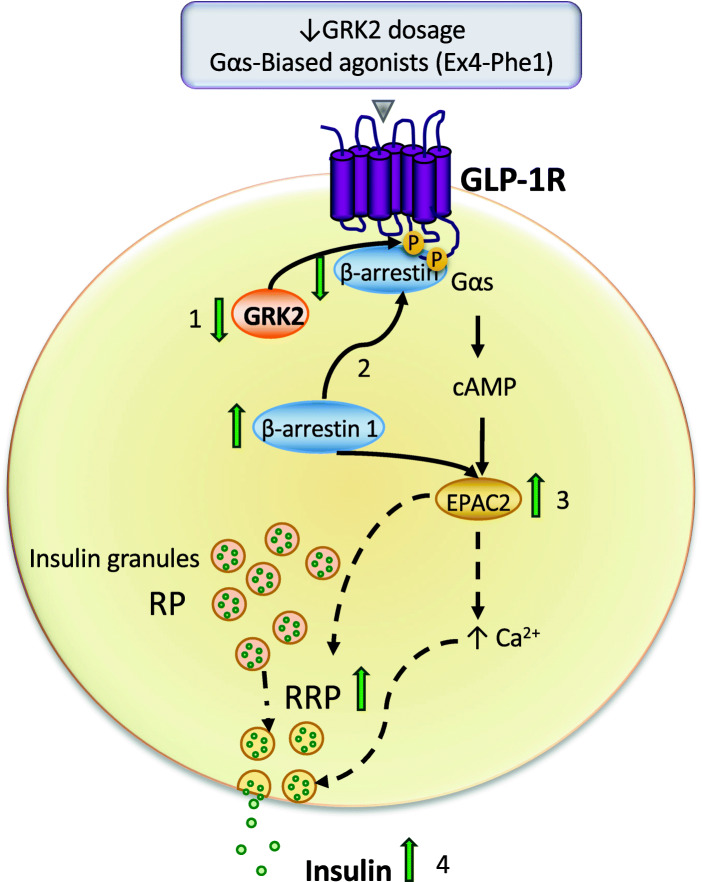
Schematic representation of the proposed impact of GRK2 on GLP-1R actions in the β-cell. Upon GLP-1R activation in the β-cell GRK2 is recruited to the activated receptor (1). In a situation of reduced GRK2 dosage or upon stimulation with Gαs-biased agonists (Ex-Phe1), less GRK2 would associate with GLP-1R leading to diminished β-arrestin recruitment (2) and reducing receptor desensitization. Also, these higher levels of free β-arrestin 1 could then activate EPAC2 (3), which would potentiate insulin secretion (4) in a dual manner: contributing to GLP-1R-mediated acute actions on insulin release (Ca^2+^), as well as increasing the size or efficiency of the RRP. Dotted lines represent indirect mechanisms of action

Overall, our data reveal a physiological role for GRK2 as a regulator of incretin-mediated insulin secretion in vivo, by modulating the GLP-1R pathway and RRP functionality (see scheme in Fig. 6). Early phase insulin release by feeding and oGTT (but not ipGTT) was enhanced in animals with reduced GRK2, as was Exendin-4-induced insulin release in vivo and in isolated islet what supports that GRK2 regulates incretin-mediated responses.

Previous reports described that GRK2 may regulate GLP-1R using model systems. For instance, in HEK293 cells, GRK2 was demonstrated to interact with the GLP-1R by BRET [47, 48] and by FRET [49]. However, a functional implication of GRK2 in the regulation of GLP-1R in a physiologically relevant in vivo context had not been addressed to our knowledge. Our data show that in pancreatic β-cell lines, GRK2 interacts with the activated GLP-1R and that downregulation of GRK2 levels or pharmacological inhibition of its activity markedly impairs β-arrestin2 recruitment to the agonist-stimulated receptor without apparent changes in Gαs recruitment.

Of note, GRK2 is recruited to a different extent by the differently biased Exendin-4 agonists. Single amino acid N-terminal modifications in Exendin-4 can bias GLP-1R agonism, and substituting histidine for phenylalanine in position 1, as in Ex4-Phe1, promotes a marked reduction in β-arrestin 1 and 2 recruitment. This leads to decreased GLP-1R degradation and increased chronic insulin secretion that is also observed upon silencing of β-arrestin proteins [45]. Interestingly, our results show that Gαs-biased GLP-1R agonists, such as Ex4-Phe1, promote a poorer recruitment of GRK2 as compared to Exendin-4 itself or to a β-arrestin-biased GLP-1R agonist (Ex4-Asp3). This speaks in favor of a mechanism by which the amount of GRK2 in the vicinity of GLP-1R could determine the efficacy of the subsequent association of β-arrestins to the phosphorylated receptor. The extent of recruitment of GRK2 would in turn depend on the agonist used (and the specific receptor conformation) as well as on the amount of GRK2 in the β-cell.

The absence of differences in Gαs-GLP-1R recruitment with different levels or activity of GRK2 despite decreased β-arrestin recruitment in such conditions could be a surprising observation that might be explained by several facts. Plasma membrane-tethered G proteins have been shown to be basally preassembled with GPCRs in some cases [50, 51], in contrast to cytoplasmic GRK2 or β-arrestins, that need to be recruited to the plasma membrane upon GPCR stimulation. Also, inactive G proteins may persist in the vicinity of GPCRs [52]. Furthermore, even when classical β-arrestin recruitment to GPCRs sterically blocks G protein binding, a ternary complex (termed “megaplex”) formed by certain class B GPCRs, a G protein and β-arrestin has been described to mediate sustained signaling in endosomal compartments [53, 54], and endosomal Gαs-signaling has been described from GLP-1R [55–57]. Pre-existing membrane complexes, permanence of inactive G protein in the vicinity of GPCRs as well as co-association of Gαs and β-arrestin to the GLP-1R would be consistent with the discrete fold-increase in exendin-mediated Gαs recruitment observed compared to that of β-arrestin2 and might contribute to explain the observed independence of Gαs association from the recruitment of GRK2 and binding of β-arrestin, at least in model experimental systems involving overexpression of receptor and partner proteins.

In any case, our data suggest that GRK2 levels and activity are critical to efficiently recruit β-arrestins to the GLP-1R. One expected outcome of this effect is the GRK2-mediated modulation of β-arrestin-mediated desensitization and thus of the canonical GLP-1R-dependent signaling pathways known to modulate insulin release. While we did not measure downstream signaling responses in β-cells after GRK2 knockdown, previous literature describes increased Gαs-mediated signaling upon GRK2 depletion or inhibition [58–61]. Moreover, GRK2 has been already shown to negatively regulate GLP-1R-stimulated cAMP production in COS7 cells [62] and also GIPR-mediated cAMP increase in a β-cell line negatively impacting insulin secretion [63].

However, it is worth mentioning here that GRK2 dosage may also modify the β-arrestin-dependent signaling cascades alternatively or in addition to the cAMP branch. The role of β-arrestins on insulin secretion remains controversial, with varying conclusions depending on the specific experimental conditions. On the one hand, β-arrestin-2 knock-out (KO) or tamoxifen-induced depletion have been shown to negatively affect glucose-mediated insulin secretion, with no apparent effect on GLP-1R-dependent responses on perfused islets [64, 65]. Conversely, islets from β-arrestin 2 KO mice show decreased levels of membrane-docked granules, what affects GLP-1R-mediated insulin secretion [66]. In turn, β-arrestin 1 KO broadly attenuates GLP-1 signaling in INS1 cells [67], while tamoxifen-induced depletion of β-arrestin 1 in mice induced no effect in Exendin-4 responses [43]. Interestingly, however, β-arrestin1 promotes insulin secretion via direct interaction with EPAC2, thus favoring EPAC2-induced Rap1 activation and potentiating sulfonylurea-induced insulin release [43]. It is thus tempting to suggest that in addition to modulating GLP-1R desensitization and thus the GLP-1R/Gs/cAMP axis, GRK2 may regulate GLP-1R-dependent insulin release by sequestering β-arrestin1 away from EPAC2 (see scheme in Fig. 6). Thus, in addition to a potential effect in fostering GLP-1R-stimulated cAMP production, decreased GRK2 levels could reduce incretin-induced GLP-1R phosphorylation and subsequent recruitment of β-arrestins, thus leaving more β-arrestin 1 available to exert its positive effects on EPAC2. Increased levels of GRK2 would promote the opposite effects. This model would also be consistent with the reported potentiation of insulin release by GLP-1R biased-agonists that trigger a reduction in β-arrestin recruitment [45, 68–70]. Compared to other experimental systems, in our in vivo conditions, neither GRK2 nor β-arrestins are completely depleted, likely reflecting a more physiological situation. Thus, we can more soundly ascribe the final enhancement of Exendin-4-mediated insulin secretion to GRK2-dependent deficiency impinging on β-arrestin recruitment in the absence of additional confounding effects.

Our data point to an implication of GRK2 in finely regulating the functionality of insulin granules. Insulin release from the β-cell occurs initially from the granules of the RRP, and subsequently by the mobilization of granules from the RP which refill the RRP [10, 71]. Interestingly, the size of the RRP directly correlates with the amount of insulin being released during the early phase [7, 71, 72]. By using arginine tolerance tests, we detect an increased size/efficiency of the RRP in GRK2 hemizygous mice in the absence of significant differences in the recruitment of vesicles from the RP. So, the increased amount of RRP insulin granules in GRK2+/− mice might explain the differences observed between genotypes in the early phase of insulin release, mostly dependent on the RRP, and also the absence of differences in the late phase, mostly dependent on the RP.

The detailed molecular mechanisms linking GRK2 to β-cell granule dynamics remain to be determined. Nonetheless, since the GLP-1R/EPAC2 pathway is a known regulator of granule dynamics [7], it is plausible to speculate that direct or indirect GRK2-dependent regulation of the GLP-1R/β-arrestin 1/EPAC2 axis is playing a role (see scheme in Fig. 6). GLP-1R-mediated potentiation of insulin secretion is required to surpass the constitutive “braking state” of the β-cell, leading to an increase in Ca^2+^ levels that is sufficient to elicit emptying of the RRP [7, 73]. This might explain the absence of differences on insulin secretion between WT and GRK2+/− mice when glucose is used as the only secretagogue, an effect that is also observed in isolated islets. Thus, a regularly enhanced response of the GLP-1R pathway, as expected to occur in GRK2+/− mice after each feeding cycle compared to WT animals, would favor more robust repetitive cycles of granule priming and transport what could be the ultimate cause for the enlarged size/functionality of the RRP observed in these animals.

Interestingly, loss of early phase insulin secretion, which our data propose to be affected by GRK2 dosage, is an early predictor of T2D onset at a time when only fasting glucose impairment has been established [74–76]. Among current treatments for T2D, GLP-1R agonists stand out as one of the most promising [55, 77–79]. Activation of the GLP-1R pathway is able to recover both the first and second phase of insulin secretion in human T2D patients [80]. Furthermore, GLP-1R agonists have a protective action on the β-cell, as well as on other tissues such as the liver and the brain [81, 82]. However, resistance to GLP-1 has been reported in diabetic patients [83, 84], which may challenge the effectiveness of treatments with GLP-1 mimetics.

Of note, previous reports have demonstrated that the level of GRK2 in different tissues is increased in situations of obesity and IR [34, 85–87]. This increase has been related with decreased insulin sensitivity and upregulation of pathology-related signaling pathways [85, 86]. Interestingly, a recent proteomics study indicated dynamic changes in islets protein levels of GRK2 in the db/db T2D mice model, with GRK2 being upregulated in hyperglycemic vs euglycemic conditions and GLP-1R displaying an opposite modulation pattern [88, 89]. Although the modulation of GRK2 levels inside the β-cell upon non-genetic IR conditions is still to be characterized, it is tempting to postulate that a potential upregulation of GRK2 could impinge on both early and late events modulated by the GLP-1R during disease progression.

## Conclusions

Taken together, our results indicate that interfering with GRK2 levels can modify GLP-1R-mediated insulin secretion in vivo specifically in the early phase which has particular interest for finding alternative or combined treatments for T2D in human patients.

## Methods

### Animal protocols

Experiments were performed using young (~ 3 months old) male C57BL/6 J (WT) mice and mice made hemizygous for GRK2 (GRK2+/−, [33]) maintained on the C57BL/6 J background. Animals were bred at a room temperature of 22 ± 2 °C on a 12:12 light–dark cycle (lights on at 08:00 am) with a relative humidity of 50 ± 10% and under pathogen-free conditions in the animal facility of the Centro de Biologia Molecular Severo Ochoa with free access to food and water. Mice were euthanized using CO_2_ or cervical dislocation, and body and pancreas weight was measured. Before group assignation, animals were weighted and distributed in the different experimental groups randomly to avoid differences in body weight. All animal experimentation procedures conformed to the European Guidelines for the Care and Use of Laboratory Animals (Directive 86/609) and were approved by the Ethical Committees for Animal Experimentation of the Universidad Autonoma de Madrid (PROEX 48/15).

#### Insulin secretion and glucose measurements

Insulin was determined in serum from blood drawn after feeding or intraperitoneal (ip) injections of Exendin-4 or sulfonylurea, and also during oral (o) and ip glucose tolerance tests (oGTT and ipGTT, respectively), or L-arginine tolerance tests (ArgGTT). Blood was extracted from the mandibular vein at the indicated time points and glucose was quantified immediately using an automatic analyzer (One Touch Ultra, LifeScan). In Fig. 5c, d, glucose was quantified using blood from the tail vein.

For the analysis of insulin levels, blood was allowed to clot after collection by leaving it undisturbed at room temperature for 30 min. The clot was removed by centrifugation at 1000*g* for 15 min in a refrigerated centrifuge, the resulting supernatant constituting the serum. Insulin content was measured in 10 μl of serum using an ELISA assay (Mouse Ultrasensitive Insulin ELISA, Mercodia).

To quantify insulin secretion after feeding, mice were fasted for 24 h and then allowed to eat standard diet pellet (Diet 150, Safe Diets*)* during the indicated time periods*.* For oGTT and ipGTTs, mice were fasted overnight for 14 h and 2 g/kg glucose (Merck, 0.2 g/ml dissolved in 0.9% NaCl) was administered by gavage or ip injection, respectively. To assess responses to GLP-1R agonists, Exendin-4 (MedChem Express, 5 μg/kg body weight dissolved in 0.2 g/ml glucose saline solution) was administered ip [20].

To study the dynamics of insulin release, L-arginine (Merck, 1 g/kg body weight dissolved in 0.9% NaCl) was injected ip in animals fasted for 14 h to depolarize the β-cell [40, 90] and thus elicit the secretion of insulin from the readily releasable pool (RRP) of insulin granules. A second ip administration of L-arginine (1 g/kg body weight) was performed 10 min after 1st ip injection to assess the replenishment of the RRP from the RP (Releasable Pool) [40].

To explore EPAC2-mediated insulin secretion mice were fasted for 1 h and injected ip with EPAC2-activating (glibenclamide, 5 mg/kg) or non-activating (glicazide, 10 mg/kg) sulfonylureas (MedChem Express) in 5% DMSO in sunflower oil [43].

#### Isolation, islet number and insulin secretion from primary murine islets

Pancreatic islets were isolated from WT or GRK2 +/− male mice by perfusion through the bile duct, as previously described [91]. Briefly, the pancreas was inflated with 5 ml of Hank’s balanced salt solution (HBSS, Ca^2+^, Mg^2+^ free, Gibco) with 0.6 mg/ml collagenase NB8 (Serva). Then, tissue was dissected and digested for 1 h at 37 °C with continuous agitation. Pancreatic islets were hand-picked and separated from the surrounding acinar tissue.

For quantification of islets number of digested WT and GRK2+/− pancreata, isolated islets were counted manually by microscopic observation after separation of the surrounding acinar tissue. Those animals, in which an incomplete perfusion of the pancreas was observed, were excluded from the final analysis (excluded mice numbers were WT *n* = 4/21 GRK2+/− *n* = 4/24).

For insulin secretion assays, isolated primary islets were cultured overnight using a 5.4-mM glucose RPMI 1640 medium. Sixteen hours after isolation, islets were transferred to Krebs-Ringer Bicarbonate Hepes (KRBH) buffer: 2 mM NaHCO_3_, 140 mM NaCl, 3.6 mM KCl, 0.5 mM NaH_2_PO_4_, 0.5 mM MgSO_4_, 1.5 mM CaCl_2_, and 10 mM Hepes, supplemented with 1% BSA and 3 mM glucose for 1 h. Subsequently, five size-matching islets were selected per technical replica and transferred to 17 mM glucose (high glucose) or 17 mM glucose + 100 nM Exendin-4 (high glucose + Ex4) KRBH to measure insulin secretion. One hour later, islets were centrifuged for 3 min at 400*g*, supernatant was conserved for secreted insulin determination and total insulin was extracted using acidified ethanol (0.135 M HCl in 75% ethanol). Insulin levels were analyzed using Mouse Insulin ELISA (Mercodia) and secreted insulin was expressed as % of total insulin content in the pancreatic islets.

#### Immunohistochemistry (IHC)

Detection of insulin (anti-Insulin Novus NBP1-19803 antibody, RRID: AB_1642425, dilution 1:200) and GRK2 (anti-GRK2 “PF2” antibody [92], dilution 1:500) was performed in mouse pancreatic tissue sections deparaffinized and rehydrated prior to antigen retrieval using citrate buffer (10 mM Sodium Citrate, 0.05% Tween 20, pH 6.0), microwave-heated to boiling temperature twice. Endogenous peroxidase activity was quenched by incubation in 3% H_2_O_2_ for 10 min before slides were washed and blocked in 5% Donkey Serum in PBS (blocking solution). Antibodies were incubated overnight at 4 °C in blocking solution including a negative control without primary antibody. Secondary antibodies (1:2000; Biotin-SP-conjugated Donkey Anti Rabbit, Jackson ImmunoResearch) were incubated in PBS for 1 h at room temperature and signal was amplified using the ABC Kit (Vector Laboratories) for 30 min. After washing, tissue sections were developed with 3,3′-diaminobenzidine (DAB) under the microscope. Finally, they were counterstained with hematoxylin and mounted with DPX (Sigma). Images were taken with a Zeiss Axioimager microscope. Pancreatic sections from tamoxifen-inducible GRK2−/− mice (Tx-GRK2−/−) animals [34] were used to assess the specificity of the GRK2 antibody (see Additional file 1: Supplementary Figure 1).

#### Quantification of β-cell mass and total insulin content

Islet β-cell mass was measured as insulin-positive areas in IHC sections separated by 400 μm to obtain a stereological analysis of the pancreas. Whole pancreatic sections were photographed at × 4 magnification (Zeiss Axioimager microscope), and all sections of the pancreas were counted, detecting an average of 30 pancreatic islets per section. Quantification of islet mass area was performed using the FIJI Software. Total insulin content of the pancreas was analyzed by acidic extraction of insulin protein as described [20]. Briefly, pancreata were dissected, sonicated in acidified ethanol (0.135 M HCl in 75% EtOH) and centrifuged at maximal speed for 1 h. Insulin was measured from the supernatant using Mouse Insulin ELISA (Mercodia).

#### Western blot

For whole-pancreas lysates, complete pancreatic tissue was homogenized in 1.5 ml of hypotonic lysis buffer as previously described for cardiac tissue [93] using metal beads in a Tissue Lyser (Qiagen) with two 2-min pulses of 1/30 s speed. Pancreatic islets and cultured cells were ruptured in lysis buffer by bath sonication and centrifuged before protein concentration was measured in the supernatant by ABC (Bio-Rad) or Lowry standard methods.

40–50 μg (whole pancreas) or 10–30 μg (cell or islets) of total protein lysates per lane were resolved by SDS-PAGE and transferred to nitrocellulose or PVDF membranes. Blots were probed with specific antibodies against GRK2 (sc-562, Santa Cruz Biotechnology, Batch number: J0615, 1:1000), β-Actin (Sigma Sigma A5441, RRID: AB_476744, Batch number: 0000088070, 1:2000), GAPDH (14C10, Cell Signaling, RRID: AB_10693448, 1:1000) and developed using enhanced chemiluminescence (ECL; Amersham Biosciences) or the Odyssey Infrared Imaging System (Li-Cor Biosciences). Films were scanned with a GS-700 Imaging Densitometer and analyzed with Quantity One Software (Bio-Rad), or using an Odyssey Classic reader and the Odyssey software package 3.0 (Li-Cor Biosciences).

#### GRK2, Gαs, and β-arrestin recruitment assays in β-cell lines

Recruitment of GRK2 to GLP-1R was measured in INS1 832/3 GLP-1R knock-out (KO; a gift from Dr Jacqueline Naylor, MedImmune, Astra Zeneca), a rat insulinoma cell line deficient for GLP-1R that was generated by CRISPR-Cas9 deletion of the GLP-1R in a INS1 832/3 background as previously described [94]. Cells were maintained in RPMI supplemented with 10% FBS, 1 mM sodium pyruvate, 10 mM Hepes, 1% penicillin/streptomycin, and 0.4% β-Mercaptoethanol. For GRK2 recruitment assays, cells were transfected with Lipofectamine 2000 with 0.5 μg GRK2-Venus (kindly provided by Dr. Meritxell Canals [95]) and 0.5 μg of a GLP-1R-NanoLuc plasmid, generated in *house* by PCR cloning of the nanoLuciferase sequence from pcDNA3.1-ccdB-Nanoluc (a gift from Mikko Taipale; Addgene plasmid # 87067) onto the C-terminus end of the SNAP-GLP-1R vector (CisBio), followed by site-directed mutagenesis of the GLP-1R stop codon. Cells were detached 24 h later and resuspended in a solution of the luminescent substrate NanoGlo® Live Cell Assay System (Promega, diluted 1:20 in HBSS). GRK2 recruitment to active GLP-1Rs was measured after the addition of 100 nM Exendin-4 by quantifying nanobioluminescence resonance energy transfer (nanoBRET) between nanoluciferase (460 nm emission) and Venus (excited at 460 nm and emitting at 535 nm) proteins [96, 97]. Recruitment was quantified during 30 min as 535 nm over 460 nm fluorescence ratio in live cells at 37 °C using FlexStation3 and the SoftMaxPro 5 software (Molecular Devices).

Min6B1 (a kind gift from Prof. Philippe Halban, University of Geneva), a clonal subline derived from the mouse insulinoma cell line Min6 [98, 99], was maintained in DMEM supplemented with 15% FBS, 1% penicillin/streptomycin, and 0.4% β-Mercaptoethanol. For Gαs recruitment [100], cells were transfected with 0.5 μg miniGs-Venus (a gift from Dr Nevin Lambert, Medical College of Georgia [52]) and 0.5 μg GLP-1R-nanoLuc plasmids, and recruitment was quantified by nanoBRET 24 h after transfection as above.

β-arrestin 2 recruitment was studied in Min6B1 cells using NanoBIT® (Promega) technology in which the two subunits of the nanoluciferase (Large (Lg)-BiT and Small (Sm)-BiT) are expressed as fusions with potentially interacting partners. Close proximity between the two subunits reconstitutes luciferase activity producing a luminescent signal, indicating partner interaction [101]. Min6B1 cells were transfected with 0.05 μg of β-arrestin-2-Lg-BiT (plasmid no. CS1603B118, Promega) and 0.05 μg of GLP-1R-Sm-BiT (generated by in-frame cloning of the SmBiT tag at the C-terminus of the GLP-1R by substitution of the Tango sequence on a FLAG-tagged GLP-1R-Tango expression vector (a gift from Dr. Bryan Roth, University of North Carolina, Addgene plasmid # 66295 [102]). Cells were resuspended in NanoGlo® Live Cell Assay System solution 24 h post-transfection to quantify luminescence. 100 nM Exendin-4 was used to activate GLP-1R and induce β-arrestin 2 recruitment. For modulation of GRK2 levels or activity, Min6B1 were transfected with Lipofectamine 2000 with 25 pmol of Scrambled (siSc; D-001810-01-05, Horizon) or αAdrbk1 siRNA (siGRK2; L-040967-00-0005, Horizon) for 48 h, or treated with the GRK2/3 inhibitor Compound 101 (30 μM, Takeda [46]) 30 min before and maintained during the assay. Luminescence was quantified during 30 min using FlexStation3 and the SoftMaxPro 5 software (Molecular Devices).

#### Data analysis

All data are expressed as mean values ± SEM and n represents the number of biological replicas (animals or independent cellular experiments) and the SEM depicts the variation in the population of study. When using cell lines or isolated islets, 2–4 technical replicas were performed in each independent experiment. Statistical significance was analyzed by using GraphPad Prism 8 and the statistic test employed is indicated in the figure legends. When comparing two data samples, data was analyzed using two-sided unpaired Student’s *t* test, or two-sided paired Student’s *t* test in cellular experiments since they were performed in parallel. Simultaneously, possible variance difference between the samples was assessed by F-test which showed no difference between groups thus validating the adequacy of the *t* test analysis. Comparisons between more than two experimental groups were performed by one-way or two-way ANOVA when the variable time is considered, since we analyzed the effect of two or more different categorical independent variables (genotype and/or treatment) on one continuous dependent variable (time). Bonferroni’s post hoc test was performed after ANOVA analysis to adjust for multiple comparisons error. Differences were considered statistically significant when *P* value < 0.05.

#### Availability of data and materials

All data generated or analyzed during this study are included in this published article and its supplementary information files (Additional File 2).

## Supplementary Information

**Additional file 1: Supplementary Figure 1**. Control of specificity of GRK2 ‘PF2’ antibody in immunohistochemistry and islets detection in WT and GRK2+/- pancreatic sections. A) Representative photomicrographs showing the immunohistochemical staining of serial pancreatic sections obtained from Tamoxifen-inducible GRK2-/- mice (Tx- GRK2-/-, Vila-Bedmar et al., 2015), WT and GRK2+/- mice using the ‘PF2’ antibody against GRK2, counterstained with hematoxylin (magnification 40x; image size adjusted to the islet area) or B) pancreas from WT and GRK2+/- mice using a 4x magnification and insulin as an islet marker (scale bar, 0.5 mm). Arrows indicate the location of the islets. Incubations without primary antibody were performed as a negative control.

**Additional file 2.** Data and results supporting the conclusions of this article.

## Data Availability

All data generated or analyzed during this study are included in this published article and its supplementary information files (Additional File 2)

## Abbreviations

Ex4: Exendin-4
EPAC2: Exchange Protein Directly Activated by cAMP 2
GLP-1: Glucagon-like peptide 1
GLP-1R: Glucagon-like peptide 1 receptor
GPCR: G protein-coupled receptor
GRK2: G protein-coupled receptor kinase 2
IR: Insulin resistance
PKA: Protein kinase A
RRP: Readily releasable pool (of insulin granules)
RP: Releasable pool
T2D: Type 2 diabetes
*Adrbk1*: Gene for GRK2
