# Supplementary material for: GRK2 regulates GLP-1R-mediated early phase insulin secretion in vivo
Source: BMC Biol. 2021 Mar 3;19:40. doi: 10.1186/s12915-021-00966-w (PMC7931601; doi:10.1186/s12915-021-00966-w)
Supplement: Supplementary file 1 — Additional file 1: Supplementary Figure 1. Control of specificity of GRK2 ‘PF2’ antibody in immunohistochemistry and islets detection in WT and GRK2+/- pancreatic sections. A) Representative photomicrographs showing the immunohistochemical staining of serial pancreatic sections obtained from Tamoxifen-inducible GRK2-/- mice (Tx- GRK2-/-, Vila-Bedmar et al., 2015), WT and GRK2+/- mice using the ‘PF2’ antibody against GRK2, counterstained with hematoxylin (magnification 40x; image size adjusted to the islet area) or B) pancreas from WT and GRK2+/- mice using a 4x magnification and insulin as an islet marker (scale bar, 0.5 mm). Arrows indicate the location of the islets. Incubations without primary antibody were performed as a negative control. [file 12915_2021_966_MOESM1_ESM.pdf]

Supplementary Figure 1

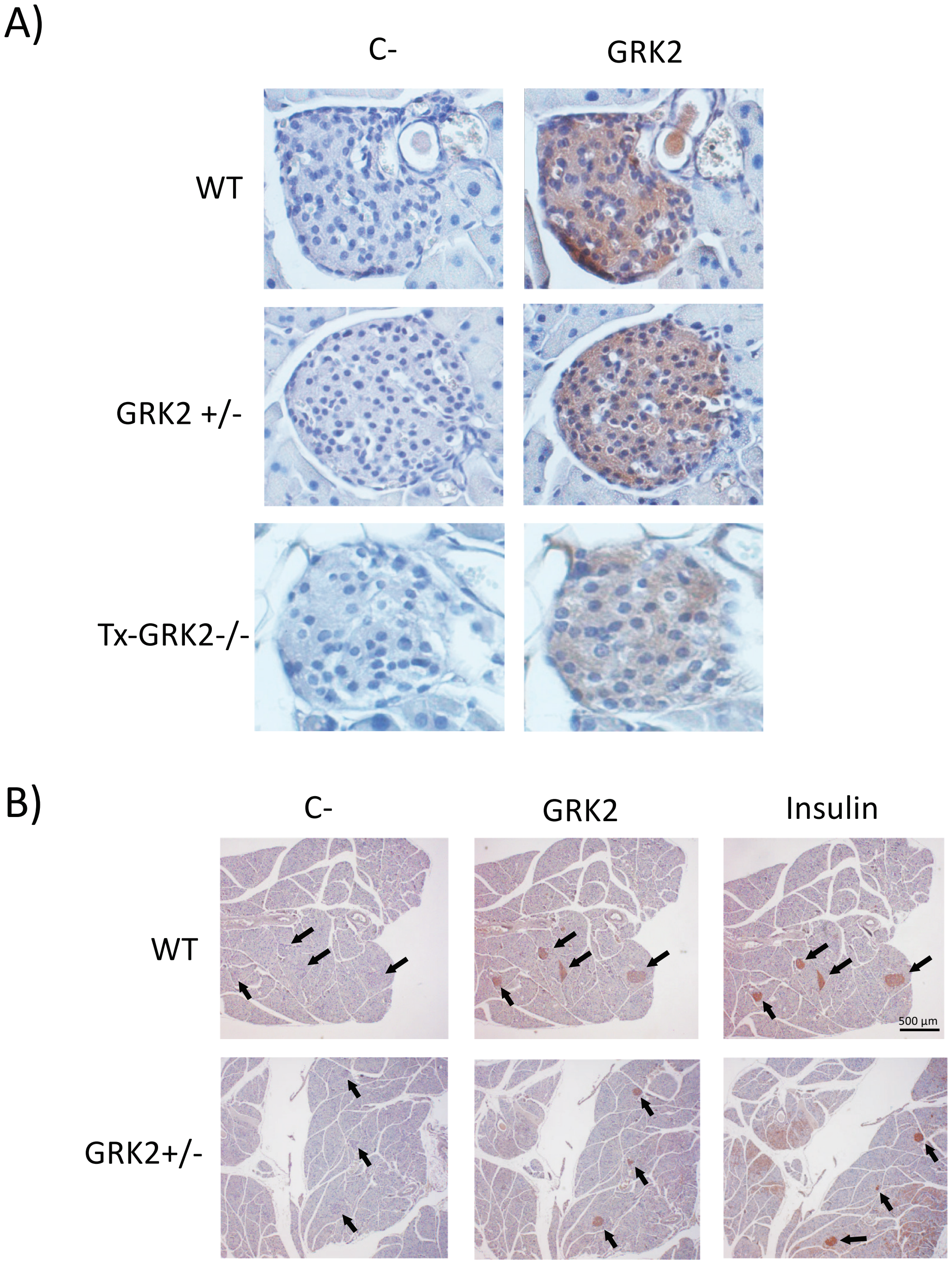

Additional File 1: Supplementary Figure 1. Control of specificity of GRK2 ‘PF2’ antibody in immunohistochemistry and islets detection in WT and GRK2+/- pancreatic sections. A) Representative photomicrographs showing the immunohistochemical staining of serial pancreatic sections obtained from Tamoxifen-inducible GRK2-/- mice (Tx- GRK2-/-, Vila-Bedmar et al., 2015), WT and GRK2+/- mice using the ‘PF2’ antibody against GRK2, counterstained with hematoxylin (magnification 40x; image size adjusted to the islet area) or B) pancreas from WT and GRK2+/- mice using a 4x magnification and insulin as an islet marker (scale bar, 0.5 mm). Arrows indicate the location of the islets. Incubations without primary antibody were performed as a negative control.
